# Using pathology-specific laboratory profiles in Clinical Pathology to reduce inappropriate test requesting: two completed audit cycles

**DOI:** 10.1186/1472-6963-12-187

**Published:** 2012-07-03

**Authors:** Roberto Baricchi, Michele Zini, Maria Grazia Nibali, Walter Vezzosi, Vincenzo Insegnante, Clotilde Manfuso, Alessandra Polese, Valmer Costoli, Antonio Spelti, Debora Formisano, Danilo Orlandini, Fausto Nicolini, Antonio Poli

**Affiliations:** 1 Clinical Pathology Department, Clinical Pathology and Transfusion Laboratory of Castelnovo nè Monti, Arcispedale Santa Maria Nuova Hospital (IRCCS), Reggio Emilia, Italy; 2 Surgical Department, Endocrinology, Arcispedale Santa Maria Nuova Hospital (IRCCS), Reggio Emilia, Italy; 3 Castelnovo né Monti Primary Care Department, Castelnovo né Monti Healthcare District, Reggio Emilia, Castelnovo nè Monti, Italy; 4 Statistics and Clinical Epidemiology Department, Statistical Unit, Arcispedale Santa Maria Nuova Hospital (IRCCS), Reggio Emilia, Italy; 5 Hospitals Healthcare Management, Reggio Emilia Health Authority, Reggio Emilia, Italy; 6 Education and Clinical Innovation Department, Healthcare Library - Clinical Governance Documentation Centre of the Healthcare Trusts of the Province of Reggio Emilia, Arcispedale Santa Maria Nuova Hospital (IRCCS), Reggio Emilia, Italy

## Abstract

**Background:**

Systematic reviews have shown that, although well prepared, the Consensus Guidelines have failed to change clinical practice. In the healthcare district of Castelnovo né Monti (Reggio Emilia, Italy), it became necessary for the GPs and Clinical Pathologists to work together to jointly define laboratory profiles.

**Methods:**

Observational study with two cycles of retrospective audit on test request forms, in a primary care setting. Objectives of the study were to develop pathology-specific laboratory profiles and to increase the number of provisional diagnoses on laboratory test request forms. A Multiprofessional Multidisciplinary Inter-hospital Work Team developed pathology-specific laboratory profiles for more effective test requesting. After 8 training sessions that used a combined strategy with multifaceted interventions, the 23 General Practitioners (GPs) in the trial district (Castelnovo nè Monti) tested the profiles; the 21 GPs in the Puianello district were the control group; all GPs in both districts participated in the trial. All laboratory tests for both healthcare districts are performed at the Laboratory located in the trial district. A baseline and a 1-year audit were performed in both districts on the GPs’ request forms.

**Results:**

Seven pathology-specific laboratory profiles for outpatients were developed. In the year after the first audit cycle: 1) the number of tests requested in the trial district was distinctly lower than that in the previous year, with a decrease of about 5% (p < 0.001); 2) the provisional diagnosis on the request forms was 52.8% in the trial district and 42% in the control district (P < 0.001); 3) the decrease of the number of tests on each request form was much more marked in the trial district (8.73 vs. 10.77; p < 0.001).

**Conclusions:**

The first audit cycle showed a significant decrease in the number of tests ordered only in the trial district. The combined strategy used in this study improved the prescriptive compliance of most of the GPs involved. The presence of the clinical pathologist is seen as an added value.

## Background

In 1995, McDonald and Smith stated that the clinical pathologist “must add value and medical relevance to the healthcare system to earn and maintain” an important role. [1] As the last 20 years have witnessed an enormous increase in laboratory medicine know-how and technology and the use of practical guidelines and laboratory profiles (a battery of tests) has increased significantly, clinical pathologists now more than ever have the opportunity to play an influential leadership role. [2] Indeed, positive experiences have highlighted the elements necessary to preparing a clinical practice guideline: there must be a multidisciplinary team that formulates clear and reasonable recommendations [3] after conducting a systematic review of the literature, an assessment of the quantity and quality (consistency, clinical impact, and organizational relationships) of the evidence.

Compliance with the guidelines does not automatically translate into appropriate patient care, however. The significant discordance between guidelines recommendations and what doctors actually do may indicate that guidelines are incomplete or that new evidence has made them obsolete [4].

There is a limited evidence base to support decisions about which guideline dissemination and implementation strategies are likely to be effective and efficient under different circumstances [5].

While not considered useful for solving particularly complex clinical problems, algorithms or, in our case, pathology-specific laboratory profiles are simpler than clinical guidelines as tools for guiding individual actions or decisions. Thanks to their simplicity they can be incorporated in computerized programs and produced in the form of flowcharts.

In Italy, GPs are directly responsible for prescribing outpatient laboratory tests. Approximately 30% of the outpatient laboratory tests are ordered without a definite working diagnosis; in these cases they are ordered, for example, “just to check,””to reassure my patients,” “because lab tests should be done at least once a year,” “for prevention,” and so on [6-8].

The healthcare services in the province of Reggio Emilia have proposed laboratory profiles that can be used in different ways [9].

The Clinical Pathology and Transfusion Laboratory of Castelnovo nè Monti (Reggio Emilia) performs all laboratory tests for the healthcare districts of Castelnovo nè Monti and Puianello.

The district of Castelnovo nè Monti has 33,000 inhabitants, 23 GPs, and a hospital with basic functions. The district of Puianello has about 23,000 inhabitants and 21 GPs; residents generally refer to the nearby main hospital of the province.

In 2007, a rapid increase in the number of laboratory tests ordered in a healthcare district in the province of Reggio Emilia (Castelnovo nè Monti) was observed, along with extreme prescriptive variability that was not necessarily strictly related to the variability of clinical situations. It was thus decided that the GPs and the Clinical Pathologists in this district would work together to jointly define laboratory profiles in order to reduce the number of unnecessary tests and ultimately, to allocate resources more efficiently.

The district of Castelnovo nè Monti was to be the project’s trial setting, while that of Puianello would be the control setting.

The short-term objective of the project was to develop pathology-specific laboratory profiles, a new method for requesting laboratory tests that was based on strong scientific evidence of efficacy without reducing the physicians’ power of discretion.

The main objectives of the project were to develop pathology-specific laboratory profiles and to increase the number of provisional diagnoses on laboratory test request forms, and were evaluated by means of a retrospective audit in both settings on the number of tests ordered over the same period of time, the presence of the provisional diagnosis on laboratory test request forms, and the average number of tests ordered on each GP request form.

## Methods

### Setting

In order to test the effectiveness of the pathology-specific laboratory profiles in reducing the number of inappropriate laboratory test requesting, two healthcare districts that refer to the same laboratory were chosen as the trial and the control setting.

### Study design

Observational study with two cycles of retrospective audit on test request forms, in a primary care setting.

### Intervention

In 2007 the Multiprofessional Multidisciplinary Inter-hospital Work Team was formed to evaluate the clinical laboratory test requesting habits of GPs in the Castelnovo nè Monti district. This team included GPs, clinical pathologists, hospital specialists, expert laboratory technicians, and medical statisticians.

The GPs involved prepared certain provisional diagnoses and one of the team’s members (RB) conducted a search on Medline (Pubmed), Embase, and The Cochrane Library, through the portal of the Healthcare Library - Clinical Governance Documentation Centre of the Healthcare Trusts of the Province of Reggio Emilia, using different appropriate combinations of the search terms*.*

Based on the results of this search and the evidence provided in the literature, the Multiprofessional Multidisciplinary Inter-hospital Work Team defined recommendations for the most effective use of laboratory tests in the clinical conditions selected. Pathology-specific laboratory profiles for these clinical conditions were developed and were presented and discussed with the GPs and hospital specialists of the Castelnovo nè Monti healthcare district (trial setting).

All laboratory tests for both healthcare districts are performed at the Laboratory located in the trial district.

All GPs in both districts participated in the trial.

Training sessions (8 in 2007) were organized in order to provide these GPs with the opportunity to understand the profiles and the scientific rationale underlying them, and to discuss their presumed usefulness. These training sessions were based on formal local consensus and on a combined strategy with multifaceted interventions like audit and feedback, reminders, marketing, and so on. [10].

As the Puianello district represented the control setting of this project, the GPs there received no training.

### Data collection

Baseline data on the total yearly number of test request forms and the total number of ordered tests were collected in both districts.

Thirty days were randomly selected over the course of one year on which the total number of test request forms and the total number of ordered tests were recorded (each request form may contain up to 8 tests, because the Emilia Romagna Region Healthcare system allows up to 8 tests on each request form); the request forms were then checked to see if the provisional diagnosis was present.

A year after introducing the new profiles in the Castelnovo nè Monti district, data on the number of test request forms and the total number of tests ordered were again collected in both the districts by means of the same method. The test request forms were evaluated by the administrative staff of the Clinical Pathology and Transfusion Laboratory using the grid developed by Multiprofessional Multidisciplinary Inter-hospital Work Team during the definition of the profiles. Data were immediately rendered anonymous and thus no researcher was able to identify any patient in any way whatsoever. Obtaining informed consent and the approval of the ethics committee were therefore not necessary.

The study design was approved by the Institutional Review Board and by the Primary care Board of the Reggio Emilia Health Authority.

### Data analysis

The weekly average number of the prescriptions (test request forms and ordered tests) and the percentage of how often the provisional diagnosis was indicated on the request forms were calculated. The statistical significance was calculated with the *χ*2 test for 2-dimensional arrays data.

The indicators used were:

1) Numerator: number of tests ordered during the second audit cycleDenominator: number of tests ordered during the baseline audit

2) Numerator: number of request forms with indication of provisional diagnosis during the audit periodDenominator: total number of request forms during the audit period

3) Numerator: average number of tests ordered on each GP request form during the second audit cycleDenominator: average number of tests ordered on each GP request form during the baseline audit.

We used the Consort Statement framework (applicable criteria) to write the report.

## Results

### Laboratory profiles

The following seven pathology-specific laboratory profiles for the first visit, for subsequent visits, and for day surgery patients were developed (see Table 1 for descriptions):

1. Normal Adult Profile

2. Myeloma

3. MGUS (Monoclonal Gammopathy of Undetermined Significance)

4. Active chronic hepatitis

5. Thyroid profile

6. Hypertension profile

7. Estro-progestogenic treatment profile

**Table 1:** Descriptions of the seven pathology-specific laboratory profiles

**Table**1**: Profile**	**Profile description**
1. Normal Adult Profile	An analysis of the literature shows that there is no rationale for ordering laboratory tests if there is no precise provisional diagnosis (or if a generic clinical suspicion is at least present) generated by the presence of symptoms, however vague and indistinct, such as asthenia, fever, and so on. In clinical trials in 1999 and 2005, the Japan Society of Clinical Pathology concluded that there was a great deal of diversity in the efficacy of the same profile when applied to different groups of patients, and that it was futile to order tests repeatedly in a single year. [7,13] Patients often see undergoing laboratory tests as unavoidable, even when no clinical signs are present [14]. It is the combination of guidelines, policy modifications to laboratory access, and changes in payment policies that is associated with significant reduction of the use of laboratories.[15] This profile consists of 8 parameters which examine the main organs or systems: Haemopoiesis: the haemachrome has 30 parameters useful for identifying numerous pathologies; Renal function: creatinine and complete urine test (itself containing 20 analytical parameters); Overall metabolism: the total cholesterol and its fractions (HDL and LDL), gpt, glycaemia. As we could not find any useful indications in the literature as to how often the profile should be repeated in a single year in a symptom-free subject, we set the maximum frequency for our healthcare setting at once a year.
2. Patient with Myeloma [16,17]	Haemochrome, creatinine, protein electrophoresis, calcaemia - ß2 microglobulin – albumin, to be repeated once a year or if there is a change in the clinical situation. Optional (but not useful in follow up): Serum and urine immunofixation, Immunoglobulin measuring
3. MGUS [14,18]	After initial classification Electrophoresis once per year. Reassess the patient to see if the clinical situation has changed.
4. Active chronic hepatitis	Normal Adult profile and Got as initial classification; Gpt - Got - haemachrome in monitoring; The serum tests for HBV and HCV are only performed for diagnostic purposes and are generally not repeated. [19,20]
5. Thyroid	TSH by screening (with the exception of pregnant women). Only if pathological TSH starts “Reflex Test” which include FT4/FT3 and, only in the first test, Antiperoxidase Antibodies. [21]
6. Hypertension	Normal Adult Profile (but optional)-na-k-cl-ca-uric acid; TSH: as initial classification in those diagnosed with hypertension the first time. Normally repeated only if there is a change in the clinical conditions or treatment. For monitoring we strongly recommend that only individual tests considered to be necessary are ordered (for example: creatinine, sodium, and potassium). [11]
7. Estro-progestogenic treatment	Normal Adult Profile is optional and not recommended. Evaluation of V Leiden Factor and of mutation of the gene coded for Prothrombin (G20210A) is recommended only in patients with family history or pathological history of venous thrombosis. Conducted only once before initiating drug therapy. [12]

### Clinical audit

**Figure 1:**
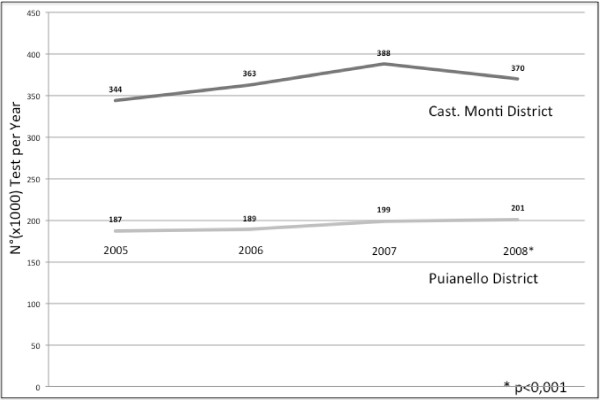
**Number of tests ordered in Castelnovo né Monti trial district (dark gray bar) and in Puianello district (light gray bar) during the course of the year, from 2005 to 2008.** The baseline audit was conducted in the year 2007 and the 2nd cycle of audits was completed in 2008.

The Clinical Pathology and Transfusion Laboratory activity data show that there was an increase in the number of tests ordered in the Castelnovo né Monti district (trial setting) between 2005 and 2007; a year after the introduction of the profiles (2008), the number of tests ordered in the same district was distinctly lower (2007:388790; 2008:370472), a decrease of about 5%. In the Puianello district (control setting), the increase was constant over the years examined, with an increase in the number of laboratory tests between 2007 and 2008 (199547 vs. 201662) of more than 1%. (Figure 1). These variations are statistically significant (p < 0.001).

In 2007 (baseline audit), there was an average of 880 laboratory test orders per week in the trial setting (Castelnovo né Monti) and an average of 642 test orders per week in the control setting (Puianello). At the end of 2008, after the training course, there was an average of 789 test orders per week in the trial setting (Castelnovo né Monti), compared to 648 test orders per week in the control setting (Puianello).

**Figure 2:**
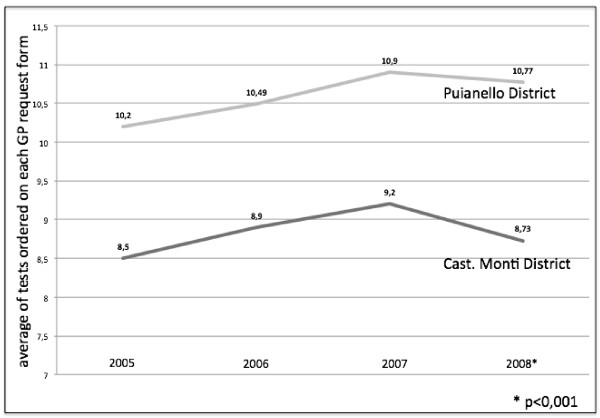
Average number of laboratory tests ordered on a single request form during the course of the year, from 2005 to 2008. Dark gray line for the Castelnovo né Monti trial district; Light gray line for the Puianello control district.

As GPs can order more than one test on a single request form, we also calculated the average number of laboratory tests ordered on each GP request form in the years between 2005 and 2008. A similar trend was seen in both districts, with an increasing ratio through 2007 and a reduction in 2008. This decrease was much more marked in the trial district (8.73 vs.10.77) (Figure 2), variations were statistically significant (p < 0.001) (Table 2).

**Table 2:** Total number of tests ordered, Weekly number of test ordered, Average of laboratory tests ordered on each GP request form, before and after the introduction of the profiles. (* p < 0,001)

**Table**2	**Baseline Audit**	**2nd Audit**
Total N° of Tests: Castelnovo nè Monti	388790	370472*
Total N° of Tests: Puianello	199547	201662*
Weekly N° of Tests: Castelnovo nè Monti	880	789*
Weekly N° of Tests: Puianello	642	648
Average of tests ordered on each GP request form: Castelnovo nè Monti	10,77	8,73*
Average of tests ordered on each GP request form: Puianello	10,90	9,20

**Figure 3:**
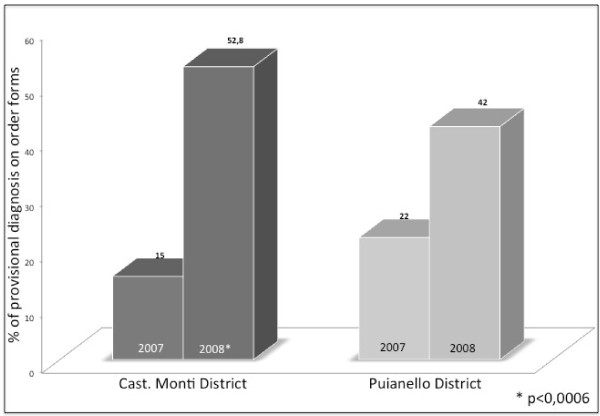
**Provisional diagnosis present on order forms.** Dark gray bars for the Castelnovo né Monti trial district; Light gray bars for the Puianello control district. The baseline audit was conducted in the year 2007, and the 2nd cycle of audit was completed in 2008.

At the baseline audit the provisional diagnosis on order forms was present in 15% of cases in the trial setting and 22% in the control setting; the difference was statistically significant (*χ*2 = 12.2, P < 0.001). After one year, the provisional diagnosis on order forms was present in about 52.8% in the trial setting and 42% in the control setting; the difference was again statistically significant (*χ*2 = 16.47 P < 0.0006) (Figure 3; Table 3).

**Table 3:** Provisional diagnosis on order forms before and after the introduction of the profiles. Data are shown as percentages (* p < 0,001; ^p < 0,0006)

**Table**3	**Castelnovo nè Monti**	**Puianello**
Provisional diagnosis: Baseline Audit	15	22 *
Provisional diagnosis: 2nd Audit	58,2	42 ^

## Discussion

The profiles were defined by means of consensus in order to facilitate joint actions. In this context clinical pathologists are extremely useful members of multidisciplinary teams that develop clinical profiles [11,12]. To minimize the differences due to the complexity of the behavioral objective defined in the study, two healthcare districts that refer to the same laboratory were chosen as the trial and the control setting. We did not plan to conduct a subsequent cross-over but only to extend the use of profiles to the Puianello district.

The laboratory test ordering habits of GPs, expressed by the number of tests per request form, measured the compliance of the GPs with the indications in the profiles. Even in the district of Puianello the number of tests per request form decreased, since at the time of the study was introduced a regional law that required doctors to write only 8 tests on each request form; however the total number of tests increased and therefore the number of request forms.

Although the pre-intervention ordering habits of GPs in the trial setting were different from those in the control district, they remained stable over time. Indeed, a significant reduction in the number of tests ordered in the first audit cycle, as compared to the baseline measurement of 2007 (about 5%), was observed only in the trial district, after application of the profiles. In contrast, a constant annual increase of about 6% was seen in the same district in the preceding years. The number of request forms that indicated a provisional diagnosis also increased significantly.

## Conclusions

An appropriate use of pathology-specific laboratory profiles resulted in a decrease in total prescriptions and a more accurate reporting of the requests, suggesting a more appropriate use of tests and a better allocation of resources, with an improvement in the cost-benefit ratio.

The combined method used in this study improved the prescriptive compliance in most of the GPs involved over a fairly short period of time, while the GPs in the control group generally maintained their previous test ordering habits.

The involvement of the clinical pathologist was deemed essential in the improvement planning stage as well as during the audits and therefore is now always present at meetings with GPs in the Castelnovo né Monti district. The intention is to involve the GPs of the Puianello district in the application of the pathology-specific laboratory profiles as well.

Audits on this approach will be conducted periodically, new profiles will be added, and existing ones will be updated. The results will continue to be discussed by the interdisciplinary work team and will be made available to all the GPs of the province of Reggio Emilia, on the Reggio Emilia Health Authority intranet.

## Competing interest

The authors declare that they have no conflict of interests.

## Author’s contributions

BR developed the format for the paper, and coordinated the collection of information and the contribution from various authors, as well as edited and formatted the final draft. FD performed the statistical analysis. BR, ZM, NMG, IV, MC, PA, CV, ST, DO, NF, PA are members of Multiprofessional Multidisciplinary Inter-hospital Work Team, and did minor editing revisions. All authors have read and approved the final manuscript.

### Funding

The resources necessary for the conduction of this study were granted by the Castelnovo né Monti Hospital.

## Pre-publication history

The pre-publication history for this paper can be accessed here:

http://www.biomedcentral.com/1472-6963/12/187/prepub
